# Entry-Level Spatial and General Non-verbal Reasoning: Can These Abilities be Used as a Predictor for Anatomy Performance in Veterinary Medical Students?

**DOI:** 10.3389/fvets.2018.00226

**Published:** 2018-09-25

**Authors:** Juan Claudio Gutierrez, Steven D. Holladay, Boaz Arzi, Marcelo Gomez, Rachel Pollard, Patricia Youngblood, Sakti Srivastava

**Affiliations:** ^1^Department of Anatomy, Physiology and Cell Biology, School of Veterinary Medicine, University of California, Davis, Davis, CA, United States; ^2^Department of Biosciences and Diagnostic Imaging, College of Veterinary Medicine, University of Georgia, Athens, GA, United States; ^3^Department of Surgical and Radiological Sciences, School of Veterinary Medicine, University of California, Davis, Davis, CA, United States; ^4^School of Veterinary Medicine, Universidad Austral de Chile, Valdivia, Chile; ^5^Department of Surgery, Division of Anatomy, School of Medicine, Stanford University, Stanford, CA, United States; ^6^Chief, Division of Clinical Anatomy, School of Medicine, Stanford University, Stanford, CA, United States

**Keywords:** spatial ability, non-verbal reasoning ability, veterinary anatomy, curriculum, anatomy

## Abstract

There is currently limited available information, but growing interest, in possible relationships between spatial visualization skills in medical students and their academic performance in select areas of the curriculum such as radiographic interpretation and anatomy. There is very limited comparable information on how entry-level spatial visualization skills may correlate with macroscopic anatomy performance in veterinary medical students exposed to an integrated curriculum. The present study made use of a battery of two short tests that measure spatial ability: Guay's visualization of views test (VVT) and mental rotation test (MRT) and, one test that measures general non-verbal reasoning abilities: Raven's Advanced Progressive Matrices Test, short form (APMT). Tests were given to 1st-year veterinary medical students (*n* = 124) immediately before commencing the integrated veterinary medical curriculum. Results show there is a positive correlation between entry-level spatial ability and non-verbal general reasoning scores confirming these abilities are linked (r: +0.22 and +0.3 for VVT/APMT and MRT/APMT respectively). The dispersion and inconsistency of significant positive correlation between anatomy practical exams grade and spatial and general reasoning scores suggest these abilities either do not correlate with anatomy practical exams grade or, are overcome with progression through the anatomy courses. Males scored higher than females in the spatial ability tests: 16.59 vs. 12.06 for VVT (*p* = 0.01) and 19.0 vs. 14.68 for MRT (*p* = 0.01). Scores for APMT did not show a significant difference by gender.

## Introduction

Gross anatomy has long been considered a keystone discipline in the medical professions. This building block portion of the medical curriculum provides the foundation for later clinical physical examinations, imaging interpretation, surgery, and pathology. A good knowledge of anatomy is also required to understand how the structure of an organism relates to its function in health. The combination of cadaver dissection, didactic lectures and supplemental materials such as textbooks and online resources, have been the traditional tools used for teaching anatomy (1).

Regarding spatial ability and anatomic learning, it has been suggested that medical students possess higher spatial ability than other science students, and also show greater improvement on spatial ability scores than students in other science disciplines after as little as 1 month of learning anatomy (2). Studies by Lufler et al. supported the idea that early intervention may be useful for students with low spatial ability when entering medical schools (3).

At the undergraduate level, Guillot et al. reported a significant positive correlation between spatial ability and academic outcome in anatomy courses. These studies did not differentiate between pre-existing spatial ability differences before the start of the anatomy course, as opposed to potential increased spatial ability occurring as a natural consequence of progressing through the highly-visual anatomy courses (4).

A companion study by Gutierrez et al. suggested that spatial and general visual reasoning abilities significantly improved in first-year veterinary medical students exposed to an integrated curriculum (5).

There are several components of spatial and visual reasoning abilities, and tests that measure such abilities. Guay's visualization of views test (VVT), and mental rotation test (MRT), are standard tests that measure spatial ability (6–8). General non-verbal reasoning ability is another skill that can be measured by standardized tests. Some of these tests employ a series of perceptual analytical reasoning problems, each in the form of a matrix (9). Raven's advanced progressive matrices test, short form, (APMT) is one such test used to measure general reasoning ability (9–11). Some general associations between spatial and reasoning abilities have been suggested. Studies by Hegarty and Kozhevnikov reveal that the use of some types of schematic spatial representations is associated with success in mathematical problem solving in sixth grade male students (12). Studies by Frydman and Lynn suggest that a combination of good general intelligence and strong spatial ability is present in gifted young Belgian chess players (13). In relation to the latter, in a study by Gliga and Iulian-Flesner, the cognitive benefit of chess training lessons was established in children. In this study, primary school students subjected to chess lessons improved their school performance in math and Romanian language (14).

There is currently information available in the literature regarding the subject of spatial visualization in human medical students. However, there is very limited information about how entry-level spatial ability and non-verbal visual general reasoning ability may predict for veterinary medical student performance.

The present study made use of a battery of three short tests that measure spatial and general reasoning abilities (VVT, MRT and APMT). This study is an extension of our 2017 report (5), in which entry-level scores from 124 students are now traced back and correlated with anatomy grades. The study was extended to these select students because this group agreed to tracking back test results to the grades of the participants: Tracking back results to correlate with grades was disclosed only with this group of students and not other groups of participants from other classes.

The hypotheses being tested were: (1) Entry-level spatial and/or general reasoning ability is correlated with performance in veterinary anatomy practical exams. Therefore, results in spatial and general reasoning tests can be used as a predictor for veterinary anatomy performance in veterinary medical students; (2) Entry-level spatial ability test results are correlated with entry-level general reasoning ability test results in veterinary medical students; and (3) There is a difference between female and male performance in entry level spatial ability tests in favor of males. However, there is no gender difference for entry-level non-verbal reasoning ability scores.

## Materials and methods

Previously validated testing tools (15) were used to evaluate entry-level spatial and general reasoning abilities in 1st–year veterinary medical students. The tests required 34 total minutes to complete, and were given to the entering class of veterinary students during their orientation week. General veterinary anatomy instruction occurs within the first 10 blocks of the integrated curriculum. Each block is 6 weeks long. The integrated curriculum at the University of California, Davis, School of Veterinary Medicine, has been designed for the adult learner and fosters the development of critical thinking, problem solving and communication skills within a comprehensive foundation of biomedical and clinical experiential learning. The curriculum provides all students with a comparative approach integrating normal and abnormal anatomy and physiology centered on body systems rather than disciplines.

Participation of first year students in this experiment was voluntary. This study was considered as exempt from formal review by the University of California Institutional Review Board. Entry level test scores from 124 students (out of 137) were analyzed. One hundred and six students were female and 18 were male.

Anatomy laboratories at the School are organized in the following structure: Three students form a dissection team. Dissection teams are responsible for a canine cadaver. The learning outcomes, lab objectives and instructions are given to the students in an online anatomy laboratory syllabus platform. Each dissection table includes a computer screen attached to the end of the table. Each dissection team receives an ipad® to access the online anatomy laboratory syllabus. The instruction of anatomy is an average of 75% labs and 25% of classroom setting.

Seven multiple choice anatomy practical exam scores were paired with entry-level scores from the three tests that measure spatial ability and visual reasoning, to evaluate possible correlations. These exams were sequential, without other interposed anatomy exams, and included a musculoskeletal midterm, musculoskeletal final, neuroscience-senses-behavior midterm, neuroscience-senses-behavior final, gastrointestinal-metabolism final, cardiovascular-respiratory final and endocrinology-reproduction final. Practical anatomy exams are station based: Anatomical structures are tagged with flags or anatomy instruments, and are displayed in multiple stations. Students have 1 min and 15 s per station. The timer is projected in two screens in the lab. After the sound of an alarm, the students move to a next station.

The testing tools for spatial and visual reasoning were provided in an online platform by Stanford University, Division of Clinical Anatomy:

1. *Guay's visualization of views test: adapted version (VVT)*: This test measures spatial ability. The test measures the ability to correctly recognize 3D objects viewed from different positions. It includes 24 questions, is timed at 8 min to complete, and is a modified and validated version of The Purdue Visualization of Views Test. (6) Questions on this test show rotated images of 3-D objects suspended in a transparent cube. The student must identify the correct corner of the cube from which a virtual picture of the suspended object was taken. The picture of the suspended object is shown above the cube in each question. Incorrect answers incur a penalty of 1/6 of a point, making the possible range of scores−4 to 24. Mean scores reported for this test in other students have been 14.3 in fourth year dentistry students, and 10.6 in undergraduate students (16, 17).
2. *Mental rotations test (MRT)*: This is a second test of spatial ability. The test requires selection of 3-D objects that are identical in shape to a reference object, but shown in different rotational orientations. This test therefore measures ability to mentally rotate complex 3-D shapes in order to find a match (7). The 40-item test is administered in two 20-item parts timed at 3 min each, for a total of 6 min. Participants receive 1 point for each correct answer and −1 point for each incorrect answer, giving a range of possible scores from −40 to 40. Average scores described by Vandenberg and Kuse for undergraduate students were: 19.06 for men and 13.17 for women (7).
3. *Raven's advanced progressive matrices test, short form (APMT)*: This test measures general non-verbal reasoning ability. This 12-question, 12-min test is a sub-set of the original full-length Raven's Advanced Progressive Matrices Test, which was validated by Bors and Stokes (10). The test requires correct identification of the missing pattern in a complex design of patterns or diagrams, from a set of 8 choices (11). Students are not penalized for incorrect answers, such that scores fall between 0 and 12. Bors & Stokes reported an overall mean score for this test of 7.39 in university students (10).

### Statistical analysis

Descriptive statistics and a Shapiro-Wilk test were performed to evaluate normal distribution. A Spearman's correlation was performed to determine if entry-level spatial ability test results (VVT and MRT) were correlated to entry-level visual reasoning test results (APMT). Spearman's correlation was also used to determine if entry-level spatial and visual reasoning test results were correlated with veterinary anatomy performance in practical exams. A Wilcoxon ranked sum test was used to compare entry-level test results from females and males. The statistical software JMP was used to perform the data analysis (JMP, Version 12, Cary, NC). For all analyses a *p* < 0.05 was considered significant.

## Results

Results from the Wilcoxon ranked sum test showed that mean scores on the GVVT were significantly different between females and males, 12.06 and 16.59 respectively (*p* = 0.0118). Mean scores on the MRT were again significantly higher in males than in females, 19.00 and 14.68 respectively (*p* = 0.0122). Scores on the RavenT did not show a significant difference between females and males, being 7.55 and 7.11 respectively (*p* = 0.362). Scores are summarized in Table 1.

**Table 1:** Mean ± SEM of female and male performance for the 3 tests of spatial and general reasoning abilities.

	**VVT (Maximum possible score: 24)**	**MRT (Maximum possible score: 40)**	**APMT (Maximum possible score: 12)**
Entry-level score for females (*N* = 106)	12.06 ± 0.52 Median: 11.5	14.68 ± 0.73 Median: 14.5	7.55 ± 0.23 Median: 8
Entry-level score for males (*N* = 18)	16.59 ± 1.93* Median: 20.5	19.00 ± 3.82* Median: 21	7.11 ± 0.6 Median: 7

* *p < 0.05*.

When performing Spearman's correlation to understand the relationship between entry-level spatial ability scores (VVT and MRT) and APMT (general reasoning) entry-level scores, a positive correlation was found between the VVT and APMT scores, and also between MRT and APMT scores. This result suggests that higher spatial ability and higher general reasoning ability in first-year veterinary students are linked skills and may be predictive for each other (Table 2).

**Table 2:** Spearman's GVVT or MRT performance, and APMT test scores.

	**VVT/APMT (Spearman p)**	**MRT/APMT (Spearman p)**
Whole group (*N* = 124)	+0.22	+0.3
Females (*N* = 106)	+0.27	+0.41
Males (*N* = 18)	+0.33	+0.03

When scores from VVT, MRT and APMT were compared to scores of the 7 anatomy practical exams using Spearman's correlation, a profile of possible associations was suggested, however was non-significant in the majority of the cases (Table 3). When analyzing results from the whole group, only a modest positive correlation was found between VVT and 4 of the practical exam scores, the other 3 practical exams didn't show any significant correlation with VVT. Scores from MRT didn't show any significant positive correlation with the anatomy practical exams outcomes. Scores from APMT showed a positive but modest correlation with only 1 of the practical exam scores, while the other 6 exam outcomes didn't show any correlation with APMT.

**Table 3:** Spearman's entry-level spatial and general reasoning test results and performance on veterinary anatomy practical exams.

	**Spearman p**
Whole group (*N* = 124)	VVT/Musculoskeletal midterm = +0.12 VVT/Neuroscience-senses-behavior midterm = +0.12 VVT/Cardiovascular-respiratory final = +0.25 VVT/Endocrinology-reproduction final = +0.10 APMT/Neuroscience-senses-behavior midterm = +0.12
Females (*N* = 106)	VVT/Musculoskeletal midterm = +0.16 VVT/Cardiovascular-respiratory final = +0.25
Males (*N* = 18)	VVT/Neuroscience-senses-behavior midterm = +0.40 VVT/Cardiovascular-respiratory final = +0.32 VVT/Endocrinology-reproduction final = +0.14 MRT Endocrinology-reproduction final = +0.22 APMT/Neuroscience-senses-behavior midterm = +0.56 APMT / Cardiovascular-respiratory final = +0.55

In females, a modest positive correlation was found between VVT and 2 of the practical exams, while the other 5 exam outcomes didn't show any correlation with VVT. Scores from MRT and APMT didn't show any correlation with anatomy practical exam outcomes in females. In males, VVT showed a modest positive correlation with 3 of the anatomy practical exam scores, and the other 4 practical exam outcomes didn't show any correlation with VVT. Scores from MRT showed a modest positive correlation with only 1 of the practical exam outcomes, and the other 6 exam outcomes didn't show any correlation with MRT. Interestingly, for APMT scores, males showed a stronger positive correlation than females with 2 of the practical exam outcomes, and the other 5 exam outcomes didn't show any correlation with APMT. Results from females, males and whole group are summarized in Table 3.

A deeper analysis was performed to compare test scores with anatomy performance: The female and male scores where divided in 2 subgroups: According to the median value for each test, each score data set was separated in “higher score subgroup”: Scores equal or higher (≥) to the median value, and “lower score subgroup”: Scores lower (<) to the median. This analysis was performed per gender for each one of the three tests. The Spearman's correlation outcomes were again inconsistent and very similar to the general data analysis. Only a few significant positive correlations where detected: For females, only one exam showed a significant positive correlation in a lower score subgroup (VVT/Cardiovascular-respiratory final: Spearman *p* = +0.28), and no significant correlations were detected for the higher score subgroups. For males, 2 significant positive correlations were detected in the lower score subgroups (MRT/Musculoskeletal final: Spearman *p* = +0.32; VVT/Endocrinology-reproduction final: Spearman *p* = +0.37). For males also, 1 significant positive correlations was detected in a higher score subgroup (MRT/Musculoskeletal final: Spearman *p* = 0.4). The rest of the associations showed non-significant positive correlations.

This may suggest that increasing the sample size by pooling data from more than one class of entering veterinary students at UC Davis would show modest but significant associations.

## Discussion

In the present study, entry-level spatial visualization ability in veterinary medical students analyzed by gender and determined by VVT and MRT showed higher scores in males. However, general reasoning ability determined by APMT did not show differences between males and females. These results are consistent with the cognitive literature. The MRT has been shown to produce one of the largest and most consistent sex differences, in favor of males (18, 19).

Studies performed by Vorstenbosh et al. reveal a significant difference in favor of male medical students in spatial ability tests. However, the males did not improve in testing with time, and, when males were excluded from the analysis, the female group scores significantly improved. Explanations for sex differences in spatial ability are still facing discrepancies in the research community. Any explanation will depend, to some extent, on when these differences first occur (18). Some researchers believe that androgen hormonal changes in puberty or, even before puberty, may be responsible for the gender differences in test performance. However, other researchers believe the changes emerge prior to adolescence (18, 20). Studies by Grimshaw et al. examined the relationship between prenatal testosterone and spatial play experiences and mental rotation performance in children. The authors support the hypotheses that testosterone may act in the fetal brain to influence the development of spatial ability (21).

In the present evaluation of general reasoning ability, using APMT, entry-level non- verbal general reasoning scores were positively correlated with entry-level spatial ability as determined by VVT and MRT. These results are supported by reports that show high spatial and general reasoning abilities may be linked with success in science and technology disciplines. Students who are above average in these two abilities, but not exceptional in mathematical or verbal skills, may be successful in science domains (22, 23). These results also correlate with studies by Keehner et al. These authors proposed that university students with high performance in both, spatial and general reasoning tests, are more rapid learners of surgical laporoscopic techniques by virtual reality than those with lower performance (24).

Due to the three dimensionality of anatomic learning, it has been suggested that academic anatomic proficiency may positively correlate with spatial ability (2, 25, 26). Studies by Lufler et al. found that medical students experienced significant visual spatial benefits during participation in the medical gross anatomy courses in the medical program (3). Similar benefits have been suggested for dentistry students (17).

When asking the opposite question: Can entry-level spatial and general reasoning abilities predict for performance in veterinary anatomy exams?; the results of the present study do not support the use of spatial and general reasoning tests as a predictor for anatomy practical exam performance. The dispersion and inconsistency of a significant positive correlation between grade and spatial and general reasoning scores suggest entry-level disparities are overcome with progression through the anatomy courses. Similarly, Sweeney et al. investigated correlations between spatial ability and performance in anatomy at the undergraduate level. These authors proposed that entry-level spatial ability may not be as important in undergraduate education. The authors described weak correlations between spatial ability and anatomy examination scores similar to the findings in the present study. Students with lower spatial ability may therefore not be at academic risks when it comes to learning anatomy, the authors proposed (27). In an opposite view, Provo et al. using the Purdue visualization of rotations test in veterinary medical students, found a significant correlation between spatial ability scores and performance on anatomy examinations. These authors therefore suggested that students with low spatial ability could be at increased risk of poorer academic outcome in anatomy (8). Lufler et al. likewise showed that entry-level spatial ability in medical students is linked to performance in gross anatomy. The authors proposed use of the MRT to identify students who may benefit from extra tutorial tools during their progression through anatomy (3).

The findings of this manuscript suggest that similar pooling of data from first year veterinary students at 2 or preferably more collaborating colleges of veterinary medicine may determine if a true consistent positive correlation exists. It may be, as well, that other tests of spatial, visual, or other reasoning ability, may be identified to significantly predict for anatomy exam outcome in single classes of veterinary students. In the same regard, comparing results with other groups of students such as pre-veterinary students would be a logical next step to follow. Another line of research could explore spatial abilities in radiology and surgery residents and, radiologists and surgeons with different years of experience in their field.

As stated in a companion study to this manuscript (5), a potential limitation of studies of this sort is voluntary participation of the students, participating students could have felt more confident in their spatial and visual reasoning abilities than non-participating students, possibly serving as a confounder of the results. Another limitation of this study is the small number of males in the study. Incoming classes are composed of an average of 86–90% of females in the School of veterinary medicine.

## Conclusion

The present study concluded that there is a positive correlation between entry-level spatial ability and non-verbal general reasoning scores suggesting these abilities are linked. The dispersion and inconsistency of significant positive correlation between grade and spatial and visual reasoning scores suggest these abilities either do not correlate with grade or are overcome with progression through the anatomy courses. There is a significant difference by gender in favor of males in entry-level spatial ability scores but not for non-verbal general reasoning scores.

## Author contributions

JG: writing, data analysis, discussion organization; SH: writing and discussion organization; BA, MG and RP: review and organization of the document; PY and SS: Review, organization of the document and provided the three used spatial ability tests online platform.

### Conflict of interest statement

The authors declare that the research was conducted in the absence of any commercial or financial relationships that could be construed as a potential conflict of interest. The handling editor declared a shared affiliation, though no other collaboration, with several of the authors JG, BA and RP.
